# GSCs in the Transdifferentiation Phenomenon: Focus on CAR-T-Based Therapy

**DOI:** 10.3390/cells15040363

**Published:** 2026-02-18

**Authors:** Martina Di Marco, Alessandro Lo Giudice, Francesca Chiara Cecala, Sabrina David, Celeste Caruso Bavisotto, Claudia Campanella, Alessandra Maria Vitale, Giuseppa D’Amico

**Affiliations:** 1 Institute of Human Anatomy and Histology, Department of Biomedicine, Neurosciences and Advanced Diagnostics (BiND), University of Palermo, 90133 Palermo, Italy; martina.dimarco@unipa.it (M.D.M.); alessandro.logiudice03@unipa.it (A.L.G.); francescachiara.cecala@community.unipa.it (F.C.C.); celeste.carusobavisotto@unipa.it (C.C.B.); alessandramaria.vitale@unipa.it (A.M.V.); giuseppa.damico01@unipa.it (G.D.); 2 Department of Precision Medicine in Medical, Surgical and Critical Area (MePreCC), University of Palermo, 90127 Palermo, Italy; sabrina.david@unipa.it; 3 Euro-Mediterranean Institute of Science and Technology (IEMEST), 90139 Palermo, Italy

**Keywords:** GBM, GSCs, transdifferentiation, CAR-T therapy

## Abstract

Glioblastoma (GBM) remains one of the most lethal brain tumors, largely due to the resilience and plasticity of glioblastoma stem cells (GSCs), which drive tumor growth, recurrence, and resistance to conventional therapies. A key mechanism underlying their aggressiveness is transdifferentiation, whereby GSCs acquire endothelial- and pericyte-like phenotypes, promoting neovascularization and remodeling the tumor microenvironment to sustain malignancy. Conventional treatments often fail to eliminate these resilient populations, highlighting the need for innovative targeted strategies. Chimeric antigen receptor (CAR)-based immunotherapies offer a targeted strategy to specifically eliminate GSCs and interfere with their role in promoting tumor vascularization and suppressing immune responses. This review aims to provide a comprehensive overview of the molecular mechanisms driving GSC transdifferentiation and to summarize the current landscape of CAR-T therapies developed to target these cells. By integrating knowledge of GSC biology with advances in CAR-T-based interventions, this work highlights the potential of next-generation immunotherapies to overcome therapeutic resistance, limit tumor recurrence, and improve clinical outcomes in GBM.

## 1. Introduction

Gliomas are among the most aggressive intracranial tumors, accounting for approximately 81% of all malignant brain neoplasms [1]. They can arise in various regions of the central nervous system (CNS) but predominantly develop in the brain, originating from glial cells or their precursors, including neural stem cells (NSCs) and astrocyte progenitors [1].

In recent years, the classification of CNS tumors has undergone a major revision with the publication of the fifth edition of the World Health Organization Classification of Tumours of the Central Nervous System (WHO CNS5) in 2021 [2]. This edition represents a substantial evolution from the 2016 version, integrating significant advances from the recommendations of the Consortium to Inform Molecular and Practical Approaches to CNS Tumor Taxonomy—Not Official WHO (cIMPACT-NOW).

Specifically, WHO CNS5 decisively strengthens the role of molecular diagnostics in defining tumor entities, moving beyond a classification based solely on histopathological criteria. In this context, glioblastoma (GBM) is now defined exclusively as an Isocitrate Dehydrogenase (IDH) wild-type diffuse glioma, characterized by specific molecular alterations such as Epidermal Growth Factor Receptor (EGFR) amplification, Telomerase Reverse Transcriptase (TERT) promoter mutation, or the +7/−10 copy number change, regardless of traditional histological grade [2]. This redefinition allows a clearer distinction between GBM and other high-grade gliomas, significantly improving diagnostic accuracy and the comparability of clinical studies.

A thorough understanding of the molecular characteristics of GBM is therefore crucial to decipher its marked biological heterogeneity, identify distinct molecular subtypes, and guide clinical decisions toward increasingly targeted, innovative, and personalized therapeutic approaches [3]. Clinically, GBM predominantly affects individuals over 40 years of age and is characterized by rapid and highly infiltrative growth, which makes complete surgical resection extremely challenging [1]. Patients may present with variable symptoms, including headache, seizures, cognitive deficits, and focal neurological impairments, often related to the location and extent of the lesion. Despite significant advances in diagnosis and therapy, prognosis remains poor: median survival is less than 15 months, and five-year survival is exceptionally low, estimated between 0.05% and 4.7% [1]. In contrast, gliomas with an oligodendroglial component generally have a more favorable clinical course than predominantly astrocytic tumors, reflecting intrinsic biological and molecular differences [1].

For high-grade gliomas (HGGs), the current standard of care consists of maximal safe surgical resection followed by concomitant radiotherapy and six weeks of temozolomide (TMZ) chemotherapy, and subsequently a six-month adjuvant phase. This regimen, known as the Stupp protocol, has significantly improved overall survival (14.6 vs. 12.1 months) and six-month progression-free survival (53.9% vs. 36.4%), as demonstrated in the randomized study by Stupp and colleagues [4].

However, despite these advances, clinical benefit remains limited, and most patients experience tumor recurrence. In addition to TMZ, other Food and Drug Administration (FDA)-approved options for HGGs treatment include lomustine, intravenous carmustine, carmustine wafers, bevacizumab, and tumor-treating fields (TTF). These approaches are mainly used in recurrent disease or for symptomatic control [5]. Among them, TTF are the only strategy that has demonstrated a significant improvement in overall survival (20.5 vs. 15.6 months) and six-month progression-free survival (56% vs. 37%) compared to standard therapy, although its use is not yet universally adopted in clinical practice [5].

A major factor underlying GBM aggressiveness and therapeutic resistance is the presence of a population of neural stem-like tumor cells, known as GBM stem-like cells (GSCs). These cells reside in specialized tumor niches and are characterized by high plasticity, self-renewal capacity, and resistance to conventional therapies, playing a key role in recurrence and disease progression [6].

GSCs can adapt to hypoxic microenvironments, modulate local immune responses, and in some cases transdifferentiate into endothelial or perivascular cells, actively participating in tumor neovascularization. Dynamic interactions between GSCs and the perivascular and hypoxic niches of GBM support stemness, invasiveness, and therapy resistance, promoting tumor evolution [6]. Overcoming GSC-mediated immune evasion mechanisms represents a major challenge in current oncology research. In this context, chimeric antigen receptor T-cell (CAR-T) therapies emerge as a promising strategy to selectively target these resistant cell populations. However, their clinical application in GBM remains limited by significant obstacles, including high antigenic heterogeneity, cellular plasticity, and the difficulty of crossing the blood–brain barrier (BBB) [7]. The following sections will discuss GSC transdifferentiation mechanisms and CAR-T strategies as potential next-generation therapies for GBM.

## 2. Vascular Transdifferentiation in GBM

GBM is a tumor characterized by a profound cellular plasticity, enabling malignant cells to undergo dynamic phenotypic rearrangements in response to intrinsic and extrinsic signals. A typical manifestation of this adaptability is the transdifferentiation phenomenon, whereby GSCs adopt lineage programs traditionally associated with non-neural cell types. Through this process, GSCs can acquire molecular and functional characteristics of endothelial cells (ECs), pericytes, and other stromal components, directly contributing to neovascularization, tumor microenvironment (TME) remodeling, and therapeutic resistance [8].

### 2.1. GSCs Transdifferentiation in ECs

One of the earliest pieces of evidence for this phenomenon was provided by Ricci-Vitiani and colleagues, who demonstrated that GSCs can differentiate into functional ECs, promoting tumor vascularization [9]. The authors observed that approximately 60% of ECs within GBM harbor the same genomic alterations as tumor cells. Thus, a substantial portion of the vasculature had a neoplastic origin. Consistently, only cultures enriched in CD133^+^ cells, i.e., GSC subpopulation, could generate CD31^+^/Tie2^+^ microvascular structures, demonstrating the stem-like compartment as the source of tumor-derived ECs (TDECs) [9]. The same authors observed that, in xenograft mouse models injected with human GSCs, nearly 70% of the vessels located within the tumor core, but not in the peripheral regions, were of human origin, further supporting the contribution of human GSCs to intratumoral vasculature [9]. Moreover, the selective targeting of these TDECs resulted in tumor reduction, underscoring this transdifferentiation pathway as both a driver of vascular heterogeneity and a potential therapeutic target [10].

According to another work, endothelial transdifferentiation of GSCs in GBM progresses through two major stages: the emergence of an endothelial progenitor-like state, and the subsequent endothelial maturation [11]. The progenitor stage represents the initial shift in GSCs toward an endothelial identity, characterized by the activation of early endothelial gene programs, whereas the maturation step involves the acquisition of functional characteristics that allow these cells to contribute effectively to the tumor vasculature [11]. The transition toward an endothelial progenitor-like state is thought to be strongly influenced by paracrine signals and microenvironmental constraints, with hypoxia acting as a central driver [11].

#### 2.1.1. Hypoxia-Dependent GSCs Transdifferentiation in ECs

One of the major events that induces GSCs to transdifferentiate into ECs is exposure to hypoxic conditions. During the early phases of tumor growth, GBM cells grow along pre-existing vessels and rely on passive diffusion for metabolic support. As proliferation accelerates, tumor cells can damage and eventually disrupt the vessels they surround, exacerbating local hypoxia. Alternatively, the expanding tumor mass may simply overcome its vascular supply, leaving peripheral regions insufficiently oxygenated. In these low-oxygen niches, GBM cells activate the hypoxia-inducible factor 1 alpha (HIF-1α) pathway, which facilitates adaptation in part through upregulation of vascular endothelial growth factor A (VEGF-A) [12]. Under hypoxic pressure, endothelial progenitors co-express both stemness-associated and endothelial markers and, during their transition into TDECs, shift from a CD133^+^/CD144^+^ phenotype to a more differentiated CD31^+^/CD34^+^ profile [12].

In a hypoxia-driven environment, CD133 critically supports the maintenance of a stem-like phenotype [13]. Its overexpression has been closely associated with enhanced self-renewal and increased resistance to TMZ, mediated through activation of the c-Jun N-terminal kinase (JNK) signaling pathway and the Notch/Sonic Hedgehog (SHH) axis, respectively. Importantly, CD133 expression is markedly upregulated under hypoxic conditions [14], underscoring the connection between low-oxygen niches, the preservation of stem-like traits, and the initiation of endothelial transdifferentiation. Accordingly, it has been observed that hypoxic conditions induce HIF-1-mediated upregulation of CD133 and a disintegrin and metalloproteinase with thrombospondin motifs 1 (ADAMTS1) in U87, U251, and U373 cells [15]. Moreover, hypoxia can also drive dedifferentiation of non-stem glioma cells into cancer stem-like cells, further expanding the GSCs subpopulation [16].

Hypoxia also drives overexpression of ETS variant transcription factor 2 (ETV2), a master regulator of endothelial cell development. In GSCs, ETV2 promoted the expression of Vascular Endothelial Growth Factor Receptor 2 (VEGFR2), CD144, and CD31 in a VEGF-A–independent manner, providing a potential explanation for GBM resistance to anti-angiogenic therapies [17]. ETV2 has been shown to induce endothelial progenitor differentiation also during cardiovascular development through upregulation of SRY-Box Transcription Factor 7 (SOX7) and Tie2 [18], suggesting that its overexpression in GBM may play a central role in orchestrating endothelial transdifferentiation.

Hypoxic niches enhance extracellular adenosine concentrations, which have been linked to GSC maintenance and increased neovascularization [19]. Rocha and colleagues observed that GSCs produced higher levels of adenosine than differentiated tumor cells, and this production was further amplified under low-oxygen conditions. In parallel, hypoxia upregulated the adenosine receptor A3 (A3AR) in GSCs, whose hyperactivation stimulated pro-survival and pro-angiogenic pathways such as phosphoinositide 3-kinase/Protein kinase B (PI3K/AKT) and mitogen-activated protein kinases (MAPK). Conversely, A3AR suppression reduced the population of cells expressing endothelial markers including CD34, CD144, and von Willebrand factor (vWF), and in vivo administration of an A3AR antagonist decreased both tumor growth and neovascularization [19].

HIF-1α directly regulates prolyl 4-hydroxylase subunit alpha 1 (P4HA1), an enzyme critical for vascular wall integrity in malignant tissues. Under hypoxic conditions, P4HA1 induces CD31 expression in GSCs via collagen type VI alpha 1 chain (COL6A1), a protein involved in matrix organization and cell–matrix interactions, thereby promoting their transition toward a tumor-derived endothelial-like phenotype. Importantly, the P4HA1–COL6A1 axis establishes a mechanistic link between hypoxia-driven extracellular matrix remodeling and endothelial transdifferentiation, ultimately supporting tumor neovascularization [20].

#### 2.1.2. The Notch Pathway Involvement in GSCs Transdifferentiation in ECs

The Notch pathway plays a pivotal role in the development of numerous cell types and tissues, including neurons, and its activity is closely tied to tumor progression. In fact, enhanced Notch signaling promoted GBM growth, whereas its inhibition reduced cancer cells proliferation and survival [21,22]. It was seen that in neurospheres derived from human GBM specimens, the pharmacological suppression of Notch signaling with γ-secretase inhibitors not only slowed tumor progression, but also prolonged the survival of mice bearing intracranial xenografts, while reducing the proportion of cells expressing the stem/progenitor markers CD133 and NESTIN [22]. Strikingly, γ-secretase-dependent inhibition also suppressed the transition from CD133^+^/CD144^−^ cells to Double Positive endothelial progenitor populations, although it did not impede their subsequent maturation into CD105^+^ TDECs, indicating that Notch signaling is particularly critical for the early progenitor phase of endothelial transdifferentiation [11].

In primary GBM samples, endothelial cells and some tumor cells express Notch ligands in proximity to NESTIN- and Notch receptor-positive GSCs [23,24]. Knockdown of the ligands involved in the Notch signaling pathway, such as Jagged canonical Notch ligand (JAG) or Delta-like canonical Notch ligand (DLL) in endothelial cells, decreases the CD133^+^ subpopulation in GBM neurospheres, highlighting the role of endothelial cells as a niche that provides Notch ligands to activate Notch signaling in GSCs [24,25]. In support of this, disruption of this pathway can drive GSCs from a stem-like state toward a more differentiated and less malignant neural phenotype. Previous studies have demonstrated that synapsin III (SYN3) promoted the neuronal-like transdifferentiation of GSCs, leading to a significant suppression of their tumorigenic potential. Mechanistically, SYN3 has been shown to increase the expression of neuregulin 3 (NRG3), which competitively binds to JAG1, thereby inhibiting the canonical JAG1–Notch1 signaling pathway [26].

#### 2.1.3. The TGF-β Pathway Involvement in GSCs Transdifferentiation in ECs

Transforming Growth Factor-β (TGF-β) is a cytokine critically involved in cancer progression. At advanced stages, TGF-β signaling promotes tumor growth by enhancing invasion, angiogenesis, immune evasion, and epithelial–mesenchymal transition. In GBM, TGF-β activity is upregulated within perivascular niches and closely associated with stemness markers such as Id1, CD44, Sox4, and Sox2 [27]. Supporting this, Ware TMB et al. demonstrated that, in U87MG, U118MG, and MU41 cell lines, the TGF-β-Activin Receptor-Like Kinase 1 (ALK)-Smad1/5 signaling pathway, a key regulator of vascular growth, is a major driver of GBM progression by promoting tumor cell transdifferentiation [28]. Their study further showed that GBM cell invasion strongly depends on this process, and in vivo inhibition of Smad1/5 significantly reduced both transdifferentiation and tumor dissemination.

Using a 3D in vitro model of the GBM perivascular niche (PVN), co-cultures of LN229 cells with human brain microvascular endothelial cells (HBMECs) exhibited increased secretion of C-X-C motif chemokine ligand 12 (CXCL12) and TGF-β. In addition, a subset of LN229 cells co-expressed the endothelial marker vWF along with the glial marker Glial Fibrillary Acidic Protein (GFAP), indicating the initiation of transdifferentiation toward TDECs [29].

#### 2.1.4. Other Pathways Involved in GSCs Transdifferentiation in ECs

WNT5A is a member of the WNT family of secreted glycoproteins, well known for their roles in embryonic development, tissue homeostasis, and stem cell regulation. In GBM, WNT5A has been implicated in maintaining GSCs’ properties and promoting invasion. Substantial evidence suggests that WNT5A also contributes to the transdifferentiation of GSCs into TDECs. CD133^+^ GSCs have been shown to express DLX5, a transcriptional regulator of WNT5A, in an AKT-dependent pathway. AKT signaling additionally inhibits the WNT5A repressor PAX6, establishing an AKT/DLX5/WNT5A axis that drives transdifferentiation [30]. In the same study, the TDECs themselves were found to express WNT5A in an AKT-dependent manner. Functionally, WNT5A produced by TDECs recruits surrounding host ECs, and enhances their proliferation, thereby promoting angiogenesis and supporting glioma cell invasion [30].

A recent work has identified DUSP8 as a key regulator of GSCs transdifferentiation into TDECs. DUSP8 functions as a molecular switch: its expression supports GSC proliferation and stemness during early tumor growth, whereas its downregulation enables endothelial transdifferentiation and tumor vascularization at later stages. This process is controlled by a specific microRNA signature, including miR-1825, which directly targets DUSP8. Clinically, low DUSP8 levels correlate with increased microvascular density and poorer patient survival. Mechanistically, DUSP8 loss enhanced MAPK pathway activation (p38, ERK, JNK), promoting endothelial-like behavior, while restoring DUSP8-impaired angiogenic functions and cytokine secretion. In vivo, DUSP8 modulation significantly affects tumor growth and vascularization, confirming its central role in GBM progression [31].

Another key oncogenic driver in GBM development is chicken ovalbumin upstream promoter-transcription factor II (COUP-TFII). Wang and colleagues demonstrated that its suppression markedly inhibits glioma cell proliferation and invasion in vitro. Mechanistically, COUP-TFII promotes GBM angiogenesis by facilitating the transdifferentiation of glioma cells into vascular endothelium-like cells, as its upregulation significantly enhanced this phenotypic conversion in vitro and increased angiogenic activity in vivo [32]. In addition to its effects on tumor-derived endothelial plasticity, COUP-TFII directly regulates Angiopoietin-1 (ANPT-1) transcription in pericytes in breast and colorectal cancers [33], further supporting angiogenic processes within the TME. Beyond vascular regulation, COUP-TFII also exerts immunomodulatory functions, as its knockdown activated antigen presentation-related pathways, alleviated immunosuppression, and enhanced T cell-mediated cytotoxicity against glioma cells in vitro [32]. Collectively, these findings suggest that targeting COUP-TFII represents a promising alternative therapeutic strategy for GBM, owing to its dual anti-angiogenic and anti-immunosuppressive effects.

### 2.2. GSCs Transdifferentiation in Pericytes

Pericytes are essential regulators of vascular homeostasis, contributing to BBB maintenance, vessel maturation, and the initiation of angiogenic sprouting. Their interactions with ECs, both through direct physical contact and reciprocal paracrine signaling, are fundamental for preserving vascular stability and function. Within the TME, pericytes may reduce the efficacy of anti-angiogenic therapies and promote angiogenesis by conferring protection to ECs [34]. In addition to giving rise to TDECs, GSCs can also transdifferentiate into tumor-derived pericyte-like cells as part of the neovascularization process. It has been demonstrated that a substantial proportion of pericytes within GBM originates from GSCs [35,36].

#### 2.2.1. The TGF-β Pathway Involvement in *GSCs Transdifferentiation in Pericytes*

GSC recruitment toward ECs is guided by a Stromal Cell-Derived Factor 1/C-X-C Chemokine Receptor 4 (SDF-1/CXCR4) chemotactic gradient, which is required for their subsequent differentiation into pericyte-like cells. Notably, EC-derived TGF-β was identified as a key cytokine driving this transdifferentiation process [35]. More recently, Cheng and colleagues provided further evidence that therapeutic interventions can reinforce this plasticity by showing that the artificial hypoxic niche generated by anti-angiogenic therapy promotes the phenotypic conversion of GSCs into pericytes, thereby supporting vascular normalization and sustained tumor growth. Mechanistically, the authors identified TGF-β delivery via small extracellular vesicles (EVs) released by hypoxic GBM cells as a key driver of this process. Exposure of GSCs to these EVs led to downregulation of stemness markers such as CD133 and SOX2, alongside upregulation of pericytic markers including Platelet-Derived Growth Factor Receptor beta (PDGFR-β), CD146, Desmin, and alpha–Smooth Muscle Actin (α-SMA), in parallel with enhanced activation of the TGF-β signaling pathway [37]. Despite its role, it was shown that pericytes differentiated in the presence of TGF-β and subsequently co-cultured with ECs were not able to establish a tight BBB, supporting the idea that TGF-β is required for early-stage differentiation rather than vessel maturation [38].

#### 2.2.2. The Notch Pathway Involvement in *GSCs Transdifferentiation in Pericytes*

Notch signaling has emerged as another key regulator of both physiological and tumor-associated vasculature, influencing not only the transdifferentiation of GSCs into endothelial cells, but also the acquisition of a pericyte-like phenotype. In gliomas, inhibition of the Notch ligand DLL4 resulted in disorganized and poorly functional vessels, highlighting the importance of this pathway in vascular patterning. Accordingly, Guichet and colleagues reported that a subset of Notch1-activated GSCs acquired a pericyte-like phenotype and were closely associated with ECs, promoting the formation of larger and more structured vessels. Importantly, Notch1 activation not only induced the expression of canonical pericyte markers but also strongly upregulated multiple angiogenesis-related factors, including interleukin-8 (IL-8), placental growth factor (PLGF), and heparin-binding epidermal growth factor (HB-EGF), underscoring a direct role for Notch signaling in tumor vascular remodeling [39].

#### 2.2.3. The EGFR Pathway Involvement in *GSCs Transdifferentiation in Pericytes*

As mentioned above, EGFR is frequently amplified in GBMs and often harbors the vIII mutation, which results in constitutive activation of the receptor’s tyrosine kinase. Segura-Collar and colleagues reported that EGFRvIII, compared to wild-type EGFR (EGFRwt), promotes the formation of more robust and enlarged tumor vessels, supporting a highly compact and hyperproliferative tumor tissue. The authors concluded that this phenotype is driven by the transdifferentiation of EGFRvIII-positive GSCs into pericyte-like cells through activation of the EGFR/BMX (bone marrow and X-linked non-receptor tyrosine kinase) signaling pathway [40]. This signaling axis promoted the expression of pericyte-associated markers, including α-SMA and CD248, in tumor cells. It also induced SOX9-dependent transcriptional programs leading to upregulation of CXCR4, CX45, and TIMP1, which contribute to enhanced chemotactic recruitment of EGFRvIII-positive GSCs toward ECs [35], as well as PDGFRβ expression, required for terminal differentiation and integration into the vessel wall [40]. Disruption of this axis targeting BMX resulted in the collapse of the vessel structure and enhanced drug delivery [41].

The EGFR pathway regulates the transdifferentiation of GSCs into pericyte-like cells in a process that is also mediated by activation of Nuclear Factor kappa B (NF-kB) [42,43]. In GBM, NF-kB becomes constitutively active upon EGFRvIII expression and is involved in stemness maintenance, invasion, resistance to therapy, and angiogenesis promotion [44]. NF-kB induces Transcriptional Co-Activator with PDZ-binding motif (TAZ), a protein that promotes the mesenchymal phenotype. Through this EGFR/NF-kB/TAZ axis, GSCs acquire mesenchymal features that facilitate transdifferentiation into pericyte-like cells. In support of this, the same authors observed a marked decrease in the number of α-SMA and CD248-positive glioma cells after TAZ inhibition [42].

#### 2.2.4. Other Pathways Involved in *GSCs Transdifferentiation in Pericytes*

Another factor recently implicated in the transdifferentiation of GSCs into pericytes is the doublesex and mab-3-related transcription factor A2 (DMRTA2). This transcription factor is overexpressed in GSCs, where it plays a role in cell proliferation and self-renewal. In GBM, high levels of DMRTA2 were detected in NESTIN^+^ and α-SMA^+^ cells located in proximity to ECs, suggesting its expression in a distinct subpopulation of cells acquiring pericyte-like functions [45]. In an in vitro tube formation assay, co-culture of HUVECs with GSCs overexpressing DMRTA2 resulted in enhanced vessel stability, suggesting that DMRTA2-expressing GSCs acquire pericyte-like features that support endothelial structures [45].

**Figure 1:**
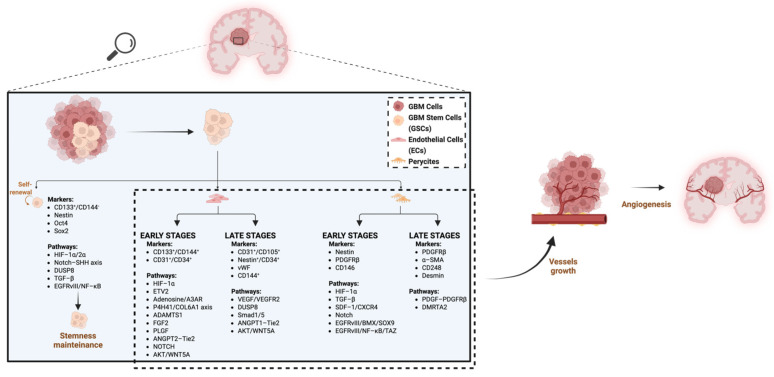
The figure illustrates the key signaling pathways driving the GSCs’ transdifferentiation into endothelial cells and pericytes during both early and late stages, and the resulting expressed markers. GSCs transdifferentiation into ECs and pericytes (dashed light blue box) contributes to the formation of new vessels within the tumor mass (curved arrow), thereby promoting angiogenesis (right-pointing straight arrow). Created in BioRender. “https://BioRender.com/oximt2n (accessed on 20 January 2026)”.

Figure 1 summarizes what was previously discussed.

### 2.3. Influence of Standard Treatments on Cellular Transdifferentiation

Although radio and chemotherapy significantly increase the overall survival (OS) of GBM patients, the treatment induces DNA damage and senescence of healthy blood vessels that may influence tumor regrowth [46]. Furthermore, therapeutic stress can activate plasticity in GBM. It has been shown that the standard treatment with TMZ and irradiation (IR) can drive GSCs towards transdifferentiation in TDECs and tumor-derived pericytes to make up for the lack of healthy vasculature [47,48,49].

Baisiwala et al. demonstrated that, in patient-derived GBM cells, treatment with TMZ increased the proportion of cells expressing the stem-cell marker CD133, as well as intermediate EC markers CD144 and CD105, and the mature EC markers CD34 and CD31. These findings were further validated in vivo in mouse models, where an expanded subpopulation co-expressing CD133 and EC markers was observed. Following TMZ administration, recurrent xenografts exhibited a significantly higher abundance of TDECs and vessels, as evidenced by increased levels of CD105 and vWF in these cells [47]. Moreover, within hypoxic perivascular niches of GBM, a subpopulation of Nestin^+^/CD31^+^ cells, derived from GSC transdifferentiation, has been shown to promote resistance to TMZ. These cells engage with GSCs to upregulate the Notch ligands JAG1 and DLL4, activating downstream effectors such as Hes1, which reinforce stemness and enhance survival under chemotherapeutic stress [23].

Following radiation, neo-angiogenesis may become less effective due to the induction of senescence. As a consequence, GSCs adopt alternative mechanisms to promote the formation of new vessels that sustain tumor regrowth. De Pascalis et al. showed a 2–2.7-fold increase in the subpopulation of tumor cells exhibiting an endothelial phenotype compared with tumors at primary surgery [48]. Deshors et al. further demonstrated that IR promoted GSCs transdifferentiation into TDECs, which displayed marked overexpression of Tie2 and its downstream effector AKT. Pharmacological inhibition of Tie2 significantly reduced the generation of TDECs from GSCs. In addition, IR induced a robust increase in TDEC migration and pseudo-tube formation, thereby enhancing their angiogenic potential, whereas Tie2 inhibition suppressed these effects. Collectively, these findings indicate that Tie2 is required for both the transdifferentiation process and the amplification of TDEC-driven angiogenesis, suggesting a pivotal role for this pathway in tumor recurrence following standard therapies [49].

It has been reported that IR exposure also increases the release of EVs from GSCs and human microvascular endothelial cells (HMVEC). In the study by Castellani G. et al., incubation of GSCs with HMVEC-derived EVs enhanced their tumorigenic properties and promoted endothelial transdifferentiation, as indicated by increased CD34 expression. Notably, these EVs were enriched in periostin (POSTN), a matricellular protein implicated in GBM angiogenesis and predominantly expressed by pericytes [50].

Lastly, it was shown that IR-induced stress promotes transdifferentiation towards both TDECs and perycite-like cells by increasing chromatin accessibility and H3K27 acetylation in specific vascular gene regions, facilitating their transcriptional activation. The histone acetyltransferase P300 mediates these epigenetic changes, and pharmacological inhibition of P300 HAT activity reverses IR-induced chromatin remodeling, suppresses vascular-like phenotype conversion, and reduces tumor growth [51].

The transdifferentiation capacity of GSCs is largely independent of VEGF signaling and is therefore not effectively targeted by anti-angiogenic therapies. Indeed, such therapies may promote tumor transdifferentiation. Inhibition of the VEGF/VEGFR pathway can induce intratumoral hypoxia, which, as previously discussed, is a major driver of the GSCs transdifferentiation process [52,53]. In line with this, the suppression of neovascularization has been shown to promote vessel co-option as an alternative vascularization strategy [54]. Wang et al. identified a CD133^+^/CD144^+^ GSCs subpopulation exhibiting features of endothelial progenitors. Notably, bevacizumab (BVZ) prevented the differentiation of tumor endothelial progenitors into mature ECs, yet did not inhibit the transition of CD133^+^ cells into endothelial precursors [11].

Anti-VEGF therapies thus appear capable of blocking the maturation of TDECs but not the initial conversion of stem-like CD133^+^/CD144^−^ cells to endothelial progenitor CD133^+^/CD144^+^. Moreover, due to the involvement of VEGF-independent pathways, transdifferentiation can still occur following prolonged exposure to these agents. Compounding this issue, GSCs typically reside in hypoxic niches with few or no functional vessels, which further restricts the effective delivery of therapeutic drugs. Ezaki et al. reported a reduction in vascular density in GBM patient-derived specimens treated with BVZ, both during and after therapy. Despite VEGF suppression, the TME initially became normoxic following BVZ administration but subsequently shifted toward hypoxia, hampering BVZ acquisition. At both early and late stages of treatment, fibroblast growth factor 2 (FGF2) and PLGF were upregulated. Additionally, ANGPT1 and ANGPT2 were elevated in the later phase. These factors-FGF2, PLGF, ANGPT1, and ANGPT2-may collectively support alternative, VEGF-independent angiogenic pathways [52]. Importantly, ANGPT2 antagonizes ANGPT1 for binding to the Tie2 receptor. ANGPT2-Tie2 signaling promotes angiogenesis by driving vessel destabilization and enhancing tumor cell migration. In light of the above, this pathway is a pivotal candidate driving this phenomenon. In support of this, Tie2 is markedly upregulated under hypoxic conditions [55] and plays a pivotal role in remodeling the TME, thereby enabling GBM cells to adopt alternative vascularization strategies.

TTF are low-intensity, intermediate-frequency alternating electric fields that disrupt mitotic processes and impair tumor cell proliferation. Emerging evidence indicates that TTF also exerts inhibitory effects on angiogenesis. Kim et al. reported a marked downregulation of HIF-1α and VEGF in GBM U87 and U373 cell lines 24 h after TTF exposure. In parallel, they observed a reduced expression of the metalloproteases MMP2 and MMP9, suggesting that TTF may also impair tumor cell migration. Moreover, TTF treatment was associated with suppression of the NF-κB, MAPK, and PI3K/AKT signaling pathways [56]. Although a direct link between TTF and the GSC transdifferentiation process has not yet been established, these findings imply that concurrent downregulation of VEGF and HIF-1α may help mitigate this phenomenon.

## 3. CAR-T-Based Immunotherapy

In recent years, immunotherapy has emerged as one of the most significant advances in modern medicine, revolutionizing the way researchers and clinicians approach the treatment of cancer and other diseases. Immunotherapy harnesses and enhances the patient’s own immune system to recognize, attack, eliminate cancer cells, and prevent tumor recurrence [57]. In this context, a wide range of strategies, including cell-based immunotherapy, inhibition of immune checkpoint pathways, development of anti-tumor vaccines, administration of oncolytic viruses, and other additional approaches, have been investigated [58]. Among these, cellular immunotherapy, particularly CAR-T cell treatment, has recently drawn substantial interest, owing to its effectiveness in treating hematologic malignancies [59]. CAR-T therapy consists of isolating T cells from a patient’s bloodstream, genetically engineering them to express synthetic antigen receptors, and reintroducing them into the patient to enable the selective targeting of tumor antigens [7]. These artificial chimeric receptors include extracellular regions designed to detect tumor-associated antigens (TAAs) and intracellular co-stimulatory signaling domains that strengthen T-cell activation. Multiple CAR-T-based treatments for liquid cancers have been authorized by the FDA based on promising initial clinical outcomes, such as the CD19-CAR-T cells for B-cell malignancies [60]. These results have thereby stimulated further studies to investigate the potential application of CAR-T therapy for solid tumors, including brain tumors [61]. In this regard, numerous preclinical studies have yielded encouraging results, paving the way for clinical trials now underway to evaluate CAR-T cell therapies for GBM and other forms of brain cancer [7,62].

### 3.1. CAR-T-Based Therapy in GBM

As explained above, GSCs constitute a small brain-resident cell subpopulation that plays a crucial role in tumorigenesis and proliferation of glioma cells in the surrounding healthy brain parenchyma [63]. Thanks to their ability to self-renew and differentiate, these cells drive tumor initiation, growth, and invasion, stimulate angiogenesis, display strong resistance to conventional therapies, and contribute to tumor recurrence [64]. In particular, treatment resistance and relapse arise from the ability of GSCs to suppress both innate and adaptive immune responses [65]. Previous studies have shown that GSCs exert a potent immunosuppressive effect on T cells [66]. Therefore, rather than focusing exclusively on classical TAAs, directing immunotherapeutic efforts toward GSCs, may represent a promising new approach for combating GBM and preventing its therapy resistance and recurrence [67]. GBM antigens have previously been targeted using antibody-based or vaccine-based strategies. However, these approaches have yielded only modest outcomes, whereas CAR-T-based therapy may offer further advantages [68]. In fact, once CAR-T cells bind to their target receptors, they can directly destroy tumor cells, making their activity independent of the endogenous immune system, which is profoundly suppressed in GBM [69]. Promising results have been obtained from clinical trials based on the use of CAR-T cells directed against EGFRvIII, interleukin 13 receptor subunit alpha 2 (IL13Rα2) and human epidermal growth factor receptor 2 (HER2) [70,71,72]. EGFRvIII results from an in-frame deletion of exons 2–7 and is present in about 30–50% of human GBM, while it is completely absent in normal tissues [73]. Clinical trials administering autologous EGFRvIII-CAR-T cells to GBM patients have demonstrated that intravenous infusion was safe and did not induce notable toxicities, including cytokine release syndrome [74]. Another study reported that combining EGFRvIII-CAR-T cell therapy with an anti-mouse VEGF antibody (B20) enhanced CAR-T cell infiltration and distribution within the GBM TME, delayed tumor progression, and extended survival in GBM-bearing mice compared to EGFRvIII-CAR-T therapy alone [70]. Another GBM-associated antigen, IL-13 receptor α-2 (IL13Rα2), is highly overexpressed in nearly all GBM tumors, while showing minimal or no expression in healthy brain tissue. Kong and coworkers described the development and evaluation of engineered T cells expressing a novel IL13Rα2-directed CAR, termed mMu IL13.CD28.ζ, demonstrating its promise as a therapeutic strategy for GBM [71]. Like IL13R α2, HER2 is also overexpressed in roughly 80% of GBM cases and has been implicated in tumor development and progression. To target this antigen, HER2-CAR-T cells were generated and tested both in vitro and in vivo, where they exhibited strong anti-tumor activity against GBM, suggesting that peritumoral administration may be a particularly effective delivery approach [72]. Recently, preclinical trials have begun assessing CAR-T cells targeting the disialoganglioside GD2 (GD2) for recurrent GBM. One example is the EchoBack-hGD2 CAR-T cell platform, designed to recognize GD2, which demonstrated potent and long-term cytotoxic capacity in 3D GBM models [75]. In mice, EchoBack-hGD2 CAR-T cells effectively suppressed GBM without inducing off-tumor toxicity and showed superior performance compared to conventional constitutively active CAR-T constructs [75].

Table 1 summarizes what was previously discussed.

However, clinical responses to CAR-T therapy in GBM vary widely among patients. Resistance to anti-cancer treatments is thought to arise from tumor heterogeneity, antigen escape mechanisms, and the highly immunosuppressive TME [76]. The substantial antigenic heterogeneity, driven by GSC plasticity, poses a challenge for effective antigen coverage by CAR-T cells, as clonal expansion of antigen-negative tumor cells or downregulation of target antigens can undermine therapeutic efficacy, even when transient antitumor responses are observed [77]. In addition, the BBB, especially in intravenous infusion therapies, restricts systemic drug penetration [78], and, yet more fundamentally, the evolved neuroimmune environment of the CNS actively constrains immune activation [79]. Multiple adverse features of the TME, that critically impair CAR-T cell function and persistence include the abundance of immunosuppressive myeloid populations and soluble inhibitory cytokines, severe metabolic constraints such as limited glucose availability and hypoxia, and the upregulation of inhibitory immune checkpoint pathways on both tumor and immune cells. Furthermore, intense competition for essential nutrients and metabolites between tumor cells and infiltrating lymphocytes restricts T cell proliferation and effector function. The TME is also characterized by the infiltration and expansion of CD8^+^ T cell-suppressive immune populations, including regulatory T cells and tumor-associated macrophages, which actively inhibit cytotoxic T cell activity through cell–cell contact-dependent mechanisms and the secretion of immunosuppressive mediators. Collectively, these hostile microenvironmental conditions create a profoundly immunosuppressive niche that limits CAR-T cell trafficking, survival, and antitumor activity [77].

Nevertheless, accumulating evidence from recent preclinical studies and clinical trials suggests that successful CAR-T therapy for solid tumors, including GBM, will depend on the integration of multiomic approaches to achieve a comprehensive understanding of the TME, encompassing genetic, epigenetic, proteomic, and metabolic dimensions, together with sophisticated genetic engineering strategies designed to exploit tumor- or patient-specific vulnerabilities [80]. Recently developed strategies to circumvent CNS-specific constraints include the design of bivalent CAR constructs targeting EGFR and IL13Rα2 to mitigate intratumoral antigen heterogeneity [81], the implementation of intracavitary and intraventricular administration routes to improve locoregional tumor access, and the generation of “armored” CAR-T cells engineered to secrete IL-15 or express dominant-negative TGF-β receptors, thereby counteracting the immunosuppressive TME [79]. In addition, the strategic incorporation of small-molecule agents and adjuvants in combination with CAR-T therapy may further enhance therapeutic efficacy [82,83]. Moreover, targeting GSCs with CAR-T cells also has the potential to address several intrinsic limitations of CAR-T therapy in GBM, including antigen heterogeneity, rapid tumor repopulation, and therapy resistance [7].

### 3.2. CAR-T-Based Therapy Against GSCs-Derived Angiogenesis

As discussed in the previous section, GSCs possess key malignant properties, including self-renewal, multilineage differentiation potential, invasive and angiogenic abilities, and pronounced resistance to therapy. Angiogenesis and invasion are well-recognized tumor-promoting behaviours triggered by hypoxic conditions, and tumors originating from GSCs are notably more vascularized and invasive than those derived from non-GSC populations [64]. Although gliomas initially sustain their growth by exploiting existing cerebral blood vessels through a process known as vascular co-option, advancing tumor expansion inevitably leads to the development of hypoxia [84]. This hypoxic microenvironment has been associated with abnormal neovascularization and correlates with significantly worse clinical outcomes [85]. In fact, tumors initiated by GSCs exhibit increased vessel density and enhanced blood perfusion compared with those generated from non-GSCs [86]. Although still debated, evidence suggests that hypoxia, including hypoxia induced by chemotherapy, may promote the transdifferentiation of GSCs into ECs, thereby contributing to the formation of tumor-derived vasculature [47]. Therefore, to inhibit GSC-derived angiogenesis, numerous efforts have been made to identify GSC-specific surface antigens for the development of GSC-targeted CAR-T cell therapies.

In one such study, researchers utilized a human surface protein antibody array and found that all validated patient-derived GSCs consistently and specifically expressed CD97 [62]. CD97 was strikingly enriched on GSCs surface, correlated with poor clinical outcomes and prognosis in GBM patients, and promoted aggressive tumor progression both in vitro and in vivo [87]. Its expression also showed a strong positive relationship with established intracellular GSC markers, including Nestin, Oct3/4, and Nanog, highlighting its potential as a highly selective surface marker for GSCs [62]. In addition, CD97 expression was significantly correlated with sphere-forming capacity, a hallmark of stemness [62]. Functionally, CD97 contributes not only to enhanced cell adhesion through interactions with extracellular matrix components but also to increased angiogenesis and tumor invasiveness [62]. Based on these insights, CD97-targeted CAR-T helper 9 (Th9) cells have been generated and demonstrated promising antitumor activity against GBM. Collectively, these findings support CD97 as a functional GSC-specific antigen and suggest that CD97-directed CAR-Th9 cell therapy may represent a powerful therapeutic approach for treatment-resistant GBM [62].

Another promising target for GSC-directed CAR-T therapy is glucose-regulated protein 78 (GRP78). GRP78, a member of the heat-shock protein 70 (HSP70) family, is primarily localized in the endoplasmic reticulum (ER), where it acts as a molecular chaperone involved in protein folding and assembly in normal cells [88]. In tumor cells, ER stress is markedly elevated as a result of uncontrolled proliferation, which leads to insufficient blood supply, hypoxia, nutrient deprivation, and heightened immune responses [89]. While normal cells typically express low levels of GRP78 confined to the ER, glioma cells and tumor-associated vascular endothelial cells exhibit both upregulated GRP78 expression and increased levels of cell surface GRP78 (csGRP78) [90]. Wang and colleagues demonstrated that csGRP78 represents a valuable therapeutic antigen and that CAR-T cells engineered to target it effectively eliminate GBM tumor cells and GSCs both in vitro and in vivo. These csGRP78-directed CAR-T cells killed tumor cells in an antigen-dependent manner and specifically secreted IFN-γ upon activation [91]. In a GBM xenograft mouse model, the CAR-T cells successfully infiltrated tumor tissues, reduced the GSC population, and ultimately suppressed tumor growth. These findings highlight csGRP78 as a compelling target for CAR-T cell therapy aimed at eradicating GSCs and improving GBM treatment outcomes [91].

Another study investigated a CAR-T approach targeting the erythropoietin-producing hepatoma (Eph) type-A receptor 3 (EphA3). Eph receptors are highly expressed during embryonic development and are present in nearly all tissues at that stage [92]. In GBM, EphA3 is strongly expressed in mesenchymal stromal cells within the tumor microvasculature as well as in self-renewing GSCs, where it plays a functional role in cell survival and self-renewal [93]. EphA3 has been reported to be specifically expressed within the vascular niche of GBM, correlating with a mesenchymal cell state known for its aggressive and infiltrative behavior. EphA3 expression was primarily localized to GSCs in perivascular regions and was restricted to newly formed tumor vasculature, while absent from normal endothelium [94]. Therefore, targeting EphA3 could potentially eliminate these cancer stem cells, achieving a more durable therapeutic response and emerging as a promising, safe therapeutic strategy. Furthermore, EphA3 displayed higher and more restricted expression in GBM cells compared with EphA2, which is also expressed in normal tissues, potentially reducing the risk of off-target effects with EphA3-directed CAR-T therapy [95].

Recent clinical trials have begun to evaluate the efficacy of CAR-T cells targeting human B7 homolog 3 (B7-H3) [96,97]. Also known as CD276 or B7RP-2, B7-H3 is a member of the B7 ligand family and has emerged as an ideal tumor antigen for antibody-based immunotherapy [98]. It is highly expressed with limited heterogeneity on differentiated tumor cells, cancer-initiating cells, tumor-associated vasculature, and stromal cells [99]. Consequently, targeting B7-H3 could not only directly eliminate tumor cells but also disrupt the TME and inhibit neoangiogenesis. In contrast, B7-H3 exhibits a highly restricted distribution in normal tissues and is notably absent in the CNS, making it a particularly attractive target for immunotherapy [99]. Approximately 70% of GBM patients express B7-H3, and CAR-T cells directed against this antigen are currently under evaluation for both adult and pediatric glioma [100]. Preclinical studies have demonstrated the potential of B7-H3-targeted CAR-T cells. One study reported novel B7-H3 CAR-T cells capable of eradicating B7-H3-positive tumors and maintaining persistent antitumor activity in a GBM xenograft model [101]. Another study showed that intracranial administration of CAR-T cells and CAR-NK cells targeting B7-H3 produced a significant antitumor effect against patient-derived GBM xenografts [97]. These findings highlight B7-H3 as a promising immunotherapeutic target for GBM.

**Figure 2:**
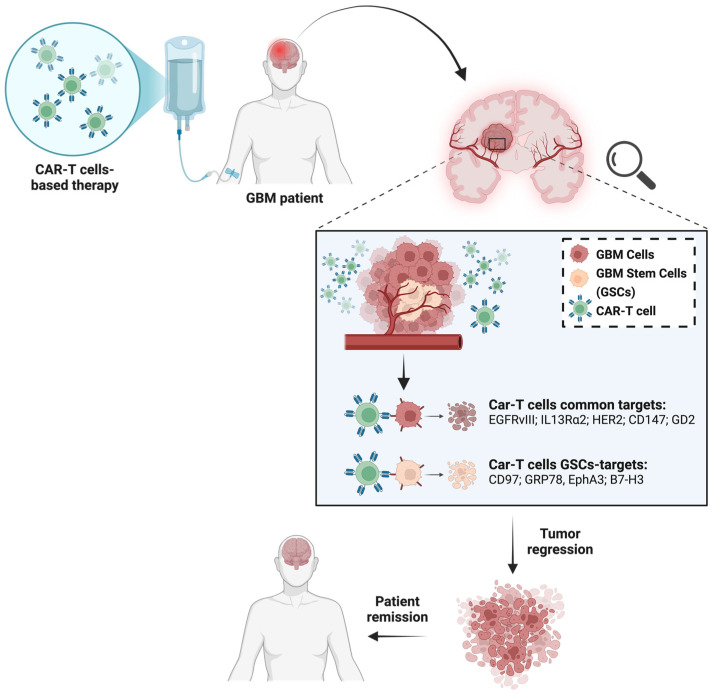
The figure shows the principal surface antigens targeted by CAR-T cells on GBM cells, as well as CAR-T targets expressed on GSCs, highlighting their contribution to tumor regression (downward straight arrow) and potential patient remission (left-pointing straight arrow). Created in BioRender. “https://BioRender.com/oximt2n (accessed on 20 January 2026)”.

Figure 2 shows CAR-T targets against both GBM cells and GSCs.

## 4. Conclusions and Future Prospects

Despite decades of research, therapeutic strategies to halt GBM progression have achieved limited clinical benefits. While targeting neoangiogenesis can restrict tumor growth, conventional anti-angiogenic therapies often fail due to alternative vascularization programs, and although CAR-T cell therapy is a powerful immunotherapeutic approach, its efficacy in GBM is constrained by the immunosuppressive TME and its limited ability to infiltrate the tumor mass and effectively reach GSCs [66].

In recent years, increasing evidence has shown that GSCs transdifferentiation represents one of the key processes involved in GBM growth and aggressiveness, playing a central role in the mechanisms driving GBM vascularization, which contributes to tumor recurrence and therapy resistance. Consequently, numerous efforts have been made to understand the underlying molecular mechanisms and the resulting findings have shifted the current perspective on this process, transforming it from a mechanism of tumor plasticity into a therapeutically exploitable vulnerability.

In this context, we propose CAR-T cell therapy as a promising approach to selectively target tumor-derived vasculature. By exploiting the molecular and genetic differences between physiological vessels and GSC-derived vascular cells, CAR-T-based strategies may overcome some of the limitations of current anti-angiogenic treatments, such as off-target effects, and open new avenues for disrupting the vascular niche that supports GBM progression. In fact, as discussed above, GSCs provide a rich repertoire of selective surface antigens that can be leveraged for targeted immunotherapies. The identification of some GSC markers, such as CD97, csGRP78, EphA3, and B7-H3 has enabled the development of sophisticated CAR-T and CAR-Th9 cell strategies capable of selectively recognizing and eliminating these therapy-resistant populations.

For instance, clinical and preclinical studies are already exploring the targeting of EGFRvIII [70,74], a receptor that is not only a well-established oncogenic driver in GBM but is also implicated in the transdifferentiation of GSCs toward a pericyte-like phenotype [40].

CD248, also known as endosialin, contributes to the maintenance of tumor microvasculature and is selectively expressed in pericytes within malignant solid tumors, including high-grade gliomas, while being largely absent in physiological brain vessels [42]. Targeting CD248 has proven to be a successful strategy in other solid tumors [102]. Similarly, the BMX non-receptor tyrosine kinase has been identified as highly expressed in GSC-derived pericytes harboring EGFRvIII mutation, but not in normal brain pericytes. Importantly, elimination of GSC-derived pericytes was shown to result in decreased expression of tight junction proteins and profound disruption of tight junction architecture in intracranial tumor vessels [41]. Disruption of tumor-derived vasculature led to increased vascular permeability, thereby enhancing the delivery of chemotherapeutic agents [41]. This observation opens the possibility of developing combinatorial therapeutic strategies involving CAR-T cell therapy. Likewise, fibroblast activation protein (FAP) has been reported to be overexpressed in GBM, together with CD90, a glioma stem cell marker, in both tumor cells and tumor-associated ECs and pericytes, supporting a potential transdifferentiation-derived origin. Notably, FAP expression is undetectable in healthy brain tissue and normal vasculature, highlighting its potential as an attractive target for CAR-T-based therapies [103].

Altogether, targeting the tumor vasculature may constitute an effective strategy to counteract tumor progression while enhancing the susceptibility of GSCs to combinatorial therapies. From a clinical perspective, investing in this new therapeutic strategy would have important implications for patient stratification and therapeutic decision-making. Targeting GSCs transdifferentiation could enable the eradication of tumour-initiating and therapy-resistant cell populations, which are largely responsible for disease recurrence, thereby supplementing current standard treatments.

## Figures and Tables

**Table 1:** The table summarizes some of the CAR-T-based therapies against targets exposed on GBM cells and the corresponding mechanism of action.

CAR-T-Base Therapy	Target	Mechanism of Action	Reference
EGFRvIII-CAR-T	Epidermal Growth Factor Receptor variant III (EGFRvIII)	Inhibition of signaling pathways such as RAS/RAF/MAPK and PI3K/AKT	[70]
mMu IL13.CD28.ζ	Interleukin 13 Receptor α-2 (IL13Rα2)	Blocking of tumor progression, invasion and metastasis	[71]
HER2-CAR-T	Human Epidermal Growth Factor Receptor 2 (HER2)	Inhibition of pathways like PI3K/Akt and MAPK	[72]
EchoBack-hGD2 CAR-T	Disialoganglioside GD2 (GD2)	Blocking of tumor growth, adhesion and immune evasion	[75]

## Data Availability

No new data were created or analyzed in this study. Data sharing is not applicable to this article.

## Abbreviations

The following abbreviations are used in this manuscript:

A3AR	Adenosine receptor A3
ADAMTS1	A disintegrin and metalloproteinase with thrombospondin motifs 1
AKT	Protein Kinase B
ALK1	Activin Receptor-Like Kinase 1
ANGPT	Angiopoietin
α-SMA	alpha–Smooth Muscle Actin
B7-H3	B7 homolog 3
BBB	Blood–Brain Barrier
BMX	Bone marrow and X-linked non-receptor tyrosine kinase
BVZ	Bevacizumab
CAR-T	Chimeric Antigen Receptor T-cell
CD	Cluster of differentiation
CNS	Central Nervous System
COL6A1	Collagen type VI alpha 1 chain
COUP-TFII	Chicken ovalbumin upstream promoter-transcription factor II
CXCL12	C-X-C motif chemokine ligand 12
DLL	Delta-like canonical Notch ligand
DMRTA2	Doublesex and mab-3-related transcription factor A2
DUSP8	Dual Specificity Phosphatase 8
ECs	Endothelial Cells
EGFR	Epidermal Growth Factor Receptor
EGFRvIII	Epidermal growth factor receptor variant III
EphA3	Erythropoietin-producing hepatoma (Eph) type-A receptor 3
ER	Endoplasmic reticulum
ERK	Extracellular signal-regulated kinase
ETV2	ETS variant transcription factor 2
EVs	Extracellular Vesicles
FAP	Fibroblast activation protein
FDA	Food and Drug Administration
FGF2	Fibroblast growth factor 2
GBM	Glioblastoma
GD2	Disialoganglioside GD2
GFAP	Glial Fibrillary Acidic Protein
GRP78	Glucose-regulated protein 78
GSCs	GBM Stem-like Cells
HB-EGF	Heparin-binding epidermal growth factor
HBMECs	Human brain microvascular endothelial cells
HER2	Human epidermal growth factor receptor 2
HGGs	High-Grade Gliomas
HIF-1α	Hypoxia-inducible factor 1 alpha
HMVEC	Human microvascular endothelial cells
HSP70	Heat-shock protein 70
IDH	Isocitrate Dehydrogenase
IL	Interleukin
IL13Rα2	IL-13 receptor α-2
IR	Irradiation
JAG	Jagged canonical Notch ligand
JNK	c-Jun N-terminal kinase
MAPK	Mitogen-activated protein kinases
MRI	Magnetic Resonance Imaging
NF-kB	Nuclear Factor kappa B
NRG3	Neuroregulin 3
NSCs	Neural Stem Cells
OS	Overall survival
P4HA1	Prolyl 4-hydroxylase subunit alpha 1
PDGFR-β	Platelet-Derived Growth Factor Receptor beta
PI3K/AKT	Phosphoinositide 3-kinase/Protein kinase B
PLGF	Placental growth factor
POSTN	Periostin
PVN	Perivascular niche
SDF-1/CXCR4	Stromal Cell-Derived Factor 1/C-X-C Chemokine Receptor 4
SHH	Sonic Hedgehog
SOX	SRY-Box Transcription Factor
SYN3	Synapsin III
TAAs	Tumor-associated antigens
TAZ	Transcriptional Co-Activator with PDZ-binding motif
TDECs	Tumor-derived ECs
TERT	Telomerase Reverse Transcriptase
TGF-β	Transforming Growth Factor-β
Th9	T helper 9
TME	Tumor microenvironment
TMZ	Temozolomide
TTF	Tumor-Treating Fields
VEGF	Vascular Endothelial Growth Factor
VEGFR2	Vascular Endothelial Growth Factor Receptor 2
WHO	World Health Organization
vWF	von Willebrand factor
